# New species of *Andropromachus* (Phasmatodea: Lonchodidae: Necrosciinae: Necrosciini) from Yunnan Province, China

**DOI:** 10.3897/BDJ.10.e78080

**Published:** 2022-01-06

**Authors:** ChongXin Xie, Jun Cai, YuHan Qian

**Affiliations:** 1 Key Laboratory for Forest Resources Conservation and Utilization in the Southwest Mountains of China, Ministry of Education, Southwest Forestry University, Kunming, Yunnan, China Key Laboratory for Forest Resources Conservation and Utilization in the Southwest Mountains of China, Ministry of Education, Southwest Forestry University Kunming, Yunnan China

**Keywords:** stick insects, external morphology, new species, taxonomy

## Abstract

**Background:**

The genus *Andropromachus* (Lonchodidae: Necrosciinae: Necrosciini) is a small genus of stick insects including four species with a distribution in northern Vietnam and south-western China.

**New information:**

A new species of stick insects *Andropromachusgulinqingensis* **sp. n.** is described from Yunnan Province of China. Diagnostic characters of the new species are illustrated and an identification key to male and female *Andropromachus* is provided along with a checklist for all described species.

## Introduction

The genus *Andropromachus*, including the two species *A.scutatus* Carl and *A.bicolor* Kirby, was erected within the tribe Necrosciini (Phasmatodea: Lonchodidae: Necrosciinae) by Carl (1913) and was later transferred to the subfamily Lonchodinae by Zompro (2001) who simultaneously designated *A.scutatus* as the type-species. Hennemann (2007) proposed that the genus *Spiniphasma* Chen & He, 2000 is a junior synonym of the genus *Andropromachus*, and therefore transferred the species *S.guangxiense* to *Andropromachus*. Hennemann (2007) further transferred *Promachustonkinensis* Brunner von Wattenwyl to *Andropromachus* as Carl (1913) had already suggested that this species may be a junior synonym of *A.bicolor* Kirby, which cannot be further examined because the type specimen of *A.tonkinensis* was lost and the original description was incomplete. Bradler et al. (2014) transferred *Andropromachus* back to Necrosciinae. *A.scutatus* was included in a recent phylogenetic study that also supported *Andropromachus* belonging to Necrosciinae (Bank and Bradler 2021).

The genus *Andropromachus* can be separated from allied genera by a combination of the following characters: convex and spinose vertex; swollen mesothorax; triangular posterolateral tooth of the abdominal tergites (Hennemann et al. 2008). At present, there are four known species in the genus *Andropromachus* throughout the world (Brock et al. 2021). Here, we provide a complete description of the new species, based on the female, male and eggs; we also give a list of all species (Table 1) and two taxonomic keys to the males and females of the genus *Andropromachus* (excluding *A.tonkinensis*).

## Materials and methods

Adults, caught in the wild, were reared in boxes and some host plants (fern) were placed inside the boxes until female oviposition. After adults and eggs were killed by low temperatures (−20℃ to ca. −40℃), adults were pinned and eggs stored in small tubes. All materials studied were deposited in the Insect Collection of the Southwest Forestry University, Yunnan Province, China (SWFU).

Morphological observations were made with a SOPTOP SZ stereomicroscope (Sunny Group Co., Ltd., China). Digital images were obtained using a Liyang Super Resolution System LY-WN-YH (Chengdu Liyang Precision Machinery Co., Ltd., China). Whole view images of the new specimens were taken with a Canon 5ds digital camera and LAOWA 100 mm F2.8 2X macro lens (Anhui Changgeng Optics Technology Co., Ltd., China). Stacking was done using the software Zerene Stacker (Zerene Systems LLC, USA, zerenesystems.com/cms/home). Morphological terminology follows that of Bragg (1997) and Bragg (2001)

## Taxon treatments

### Andropromachus gulinqingensis

Xie & Qian sp. n.

DFB7216B-DCFD-5416-AE84-268668CA5F8C

303602BC-E9B7-4934-9CF7-7DAF8289E945

#### Materials

- **Type status:** Holotype. **Occurrence:** recordedBy: Xiang-Jin Liu; sex: female; lifeStage: adults; **Taxon:** scientificName: *Andropromachusgulinqingensis*; order: Phasmatodea; family: Lonchodidae; genus: Andropromachus; **Location:** country: China; stateProvince: Yunnan Province; county: Maguan County; municipality: Wenshan Zhuang and Miao Autonomous Prefecture; verbatimLocality: Maguan County, Gulinqing Provincial Nature Reserve; verbatimLatitude: N22.81339°; verbatimLongitude: E103.97092°; **Event:** year: 2020; month: 7; day: 15; **Record Level:** institutionCode: Southwest Forestry University, Yunnan Province, China (SWFU)
- **Type status:** Paratype. **Occurrence:** recordedBy: Xiang-Jin Liu; sex: 2 females, 2 males, 10 eggs; lifeStage: adults; **Taxon:** scientificName: *Andropromachusgulinqingensis*; order: Phasmatodea; family: Lonchodidae; genus: Andropromachus; **Location:** country: China; stateProvince: Yunnan Province; county: Maguan County; municipality: Wenshan Zhuang and Miao Autonomous Prefecture; verbatimLocality: Maguan County, Gulinqing Provincial Nature Reserve; verbatimLatitude: N22.81339°; verbatimLongitude: E103.97092°; **Event:** year: 2020; month: 7; day: 15; **Record Level:** institutionCode: Southwest Forestry University, Yunnan Province, China (SWFU)

#### Description

##### Female

**Figure 1.**
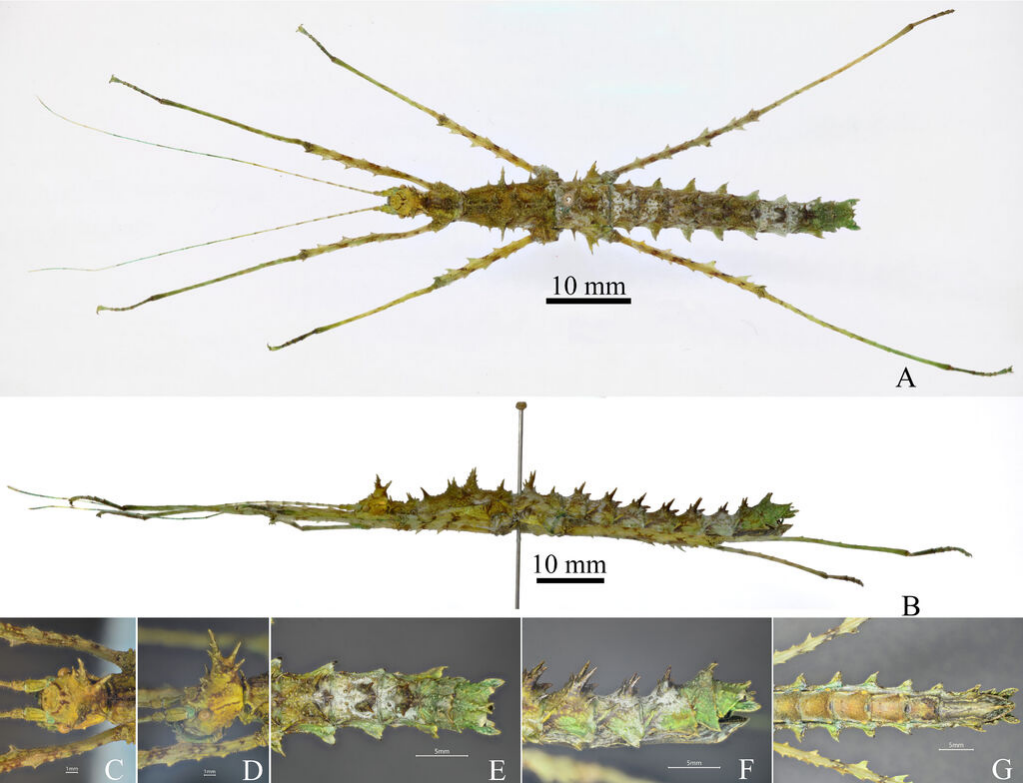
*Andropromachusgulinqingensis* sp. n., female, holotype. **A.** habitus, dorsal view; **B.** habitus, lateral view; **C.** head, dorsal view; **D.** head, lateral view; **E.** terminalia, dorsal view; **F.** terminalia, lateral view; **G.** terminalia, ventral view.

Medium size. Body robust. The general colouration of the body is green (Fig. 1A-G).

**Head.** Globose, longer than wide, vertex flat, sparsely covered with a few small granules and interspersed with a few acute small granules. Compound eyes rounded, occupying 1/4 of the genae. Antennae filiform, longer than forelegs; scapus rectangular, flattened and longer than pedicellus, pedicellus cylindrical and shorter than the third segment. Occiput prominently swollen and convex, with three pairs of spines on both sides of the median longitudinal sulci; anterior spines behind the compound eyes; median spines largest and with a few small branches; posterior spines close to median spines (Fig. 1C and D). **Thorax.** Pronotum trapezoid, as long as the head, with sparse and small granules; anterior margin slightly concave, posterior margins slightly rounded; transverse and longitudinal sulci crossing at mid-area and distinct; three pairs of spines on the two sides of median longitudinal carina, a pair of spines located at the anterior margins of pronotum, two pairs of spines located at the posterior margins of pronotum, the second pair of spines longest (Fig. 1A and B). Mesonotum trapezoid, longer than width, 2.5x length of pronotum, sparsely covered with a few small granules, median longitudinal carina distinct; five pairs of spines on the two sides of median longitudinal carina and the few spines with small branches, the first pair of spines located at anterior margins of mesonotum, the second pair of spines located at 1/3 length of mesonotum, the third pair of spines largest and located at the middle of mesonotum, the fourth pair of spines located at 2/3 length of mesonotum, the fifth pair of spines located at the posterior margin of mesonotum; four spines on the lateral carina of mesonotum and successively larger from front to back (Fig. 1A and B). Metanotum rectangle, 0.5x length of mesonotum, two pairs of spines on the two sides of median longitudinal carina, a pair of spines located at the middle of metanotum, largest and with small branches, a pair of spines located at 2/3 length of metanotum, a spine on the lateral carina of metanotum (Fig. 1A and B). **Abdomen.** Cylindrical, sparsely granulated. Median segment semicircle, wider than length, almost 1/2 length of metanotum, not obviously segmented, with two pairs of spines, a pair of spines located at 1/3 length of median segment, a pair of spines located at 2/3 length of median segment. Tergites II-IX: each segment with the posterolateral angle strongly elevated to form a prominent triangular, apically pointed lobe (Fig. 1A and B). Tergites II-VIII: each segment armed with five spines, two anterior spines located at 2/3 of tergites, largest and with branches at base, three posterior spines on the posterior margin of tergites, these spines most prominent on tergites II-VI (Fig. 1A, B, E and F). Sternite VII with a flat praeopercular organ (Fig. 1G). Tergite IX as long as anal segment, with a posteromedial crest and with two spines on the posterior margin of posteromedial crest. Anal segment with a few tubercles, median longitudinal carina distinct, two spines on the postmedian margin of anal segment, two strap-shaped bulges on posterolateral margin of anal segment (Fig. 1E, F). Subgenital plate scoop-shaped, tapering posteriorly, apex pointed and reaching posterior margin of anal segment (Fig. 1G). Cerci long, slightly lanceolate and surpassing posterior margin of anal segment (Fig. 1E, F). **Legs**: All long and moderately slender and sparsely covered with short bristles, all femora shorter and thicker than corresponding tibiae; Profemora distinctly curved basally; carinae of pro-, meso- and metafemora with distinct serrations; pro- and mesotibiae smooth and without small serrations, metatibiae with a few indistinct small serrations (Fig. 1A and B).

##### Male

**Figure 2.**
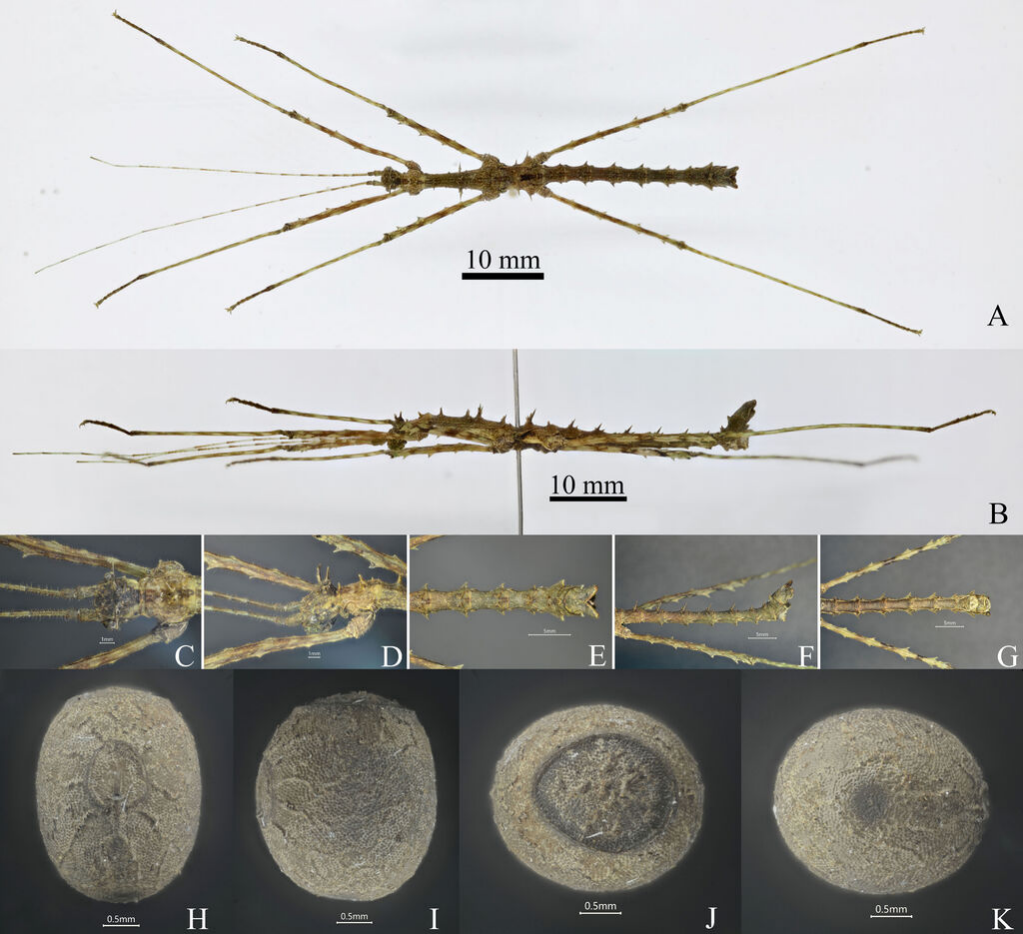
*Andropromachusgulinqingensis* sp. n., male and egg, paratype. **A.** male habitus, dorsal view; **B.** male habitus, lateral view; **C.** male head, dorsal view; **D.** male head, lateral view; **E.** male terminalia, dorsal view; **F**. male terminalia, lateral view; **G.** male terminalia, ventral view; **H.** egg, dorsal view; **I.** egg, lateral view; **J.** egg, opercular view; **K.** egg, polar view.

Small size. Body robust. The general colouration of the body is green or brown (Fig. 2A-G).

**Head.** Globose, longer than wide, vertex flat, covered with sparse and small granules and a few acute small granules. Compound eyes rounded, occupying 1/4 of the genae. Antennae filiform, longer than forelegs, scapus rectangular and flattened, longer than pedicellus, pedicellus cylindrical and shorter than the third segment. Occiput prominently swollen and convex, with two pairs of spines on both sides of the median longitudinal sulci, the anterior spines larger than posterior spines and these two pairs of spines close together (Fig. 2C and D). **Thorax.** Pronotum as long as the head, with sparse and small granules; transverse and longitudinal sulci crossing at middle area and distinct; three pairs of spines on the two sides of median longitudinal carina, the anterior spines located at the anterior margins of pronotum, the median spines largest and located at 2/3 length of pronotum, the posterior spines located at 4/5 length of pronotum and very small and indistinct (Fig. 2A and B). Mesonotum trapezoid, longer than width, 4x length of pronotum; median longitudinal carina distinct, sparsely covered with a few small granules; with five pairs of spines on the two sides of the median longitudinal carina and some spines with small branches, the first pair of spines located at 1/7 length of mesonotum, the second and third pair of spines located at 1/2 length of mesonotum, the fourth pair of spines largest and located at 2/3 length of mesonotum, the fifth pair of spines located at the posterior margin of mesonotum; two spines on the lateral carina and are successively larger from front to back (Fig. 2A and B). Metanotum rectangle, 0.3x length of pronotum; a pair of spines on the posterior margin of metanotum and with small branches at base (Fig. 2A and B). **Abdomen.** Cylindrical, sparsely granulated. Median segment square, as long as metanotum, a pair of tiny spines on the posterior margin. Tergites II-IX each with the posterolateral angle strongly elevated and forming a prominent triangular lobe and apically pointed (Fig. 2A, B, E and F). Tergites II-VIII: each segment armed with three spines, two anterior spines located at 2/3 of tergites, largest and with a small branch basally, a posterior spine on the postmedian margin of tergites, the three spines distinct on tergites II-VI (Fig. 2A, B, E and F). Tergite IX with a posteromedial crest and two tumours on the posterior margin (Fig. 2E and F). Anal segment longer than tergum IX, posterior margin with deep V-shaped emargination, posterolateral angles obtuse (Fig. 2E). Poculum cup-shaped, posterior margin rounded, reaching anterior margin of anal segment in lateral view. Vomer tongue-shaped, apices rounded and without teeth. Cerci flattened, knife-shaped, apices pointed (Fig. 2G). **Legs**: All long and moderately slender and sparsely covered with short bristles; all femora shorter and thicker than corresponding tibiae, profemora basal curved indistinctly; carinae of pro-, meso- and metafemora with distinct serrations; carinae of all tibiae smooth and without small serrations (Fig. 2A and B).

##### Egg

General colouration brown. Capsule oval, surface with sparse retirugose and densely granulose (Fig. 2H-K). Micropylar plate circular, located at the anteromedian of capsule, anterior apex margin narrowly rounded, posterior apex margin broadly rounded (Fig. 2H-J). Micropylar cup grey-black and distinct, a short light brown median line under the micropylar cup (Fig. 2H). Operculum almost circular and convex slightly and central lacking capitulum, collar distinct (Fig. 2H and I). Polar mound area rounded (Fig. 2K).

##### Measurements (mm)

**Female.** Body length 52.0-56.0; head length 3.0-4.5; pronotum length 3.5-4.0; mesonotum 11.0-12.0; metanotum 4.5-5.5; median segment 2.2-3.0; profemora 14.5-16.0; mesofemora 10.0-12.0; metafemora 18.0-20.0; protibiae 16.0-18.0; mesotibiae 14.0-15.0; metatibiae 20.0-23.0. **Male.** Body length 46.0-49.0; head length 2.5-3.0; pronotum length 3.0-3.5; mesonotum 10.0-10.5; metanotum 3.0-3.5; median segment 3.0-3.5; profemora 15.5-16.0; mesofemora 13.0-13.5; metafemora 18.0-19.0; protibiae 19.0-20.0; mesotibiae 10.5-11.0; metatibiae 22.5-23.5. **Egg.** Width 4.3-4.6, height 5.0-5.4, length 5.5-5.7.

#### Diagnosis

*Andropromachusgulinqingensis* sp. n. is similar to *A.guangxiense* (Chen & He, 2000), but can be distinguished by: ♀♀, middle size (body length > 50 mm), without spines between the compound eyes (Fig. 1C), abdominal tergites II–VIII: each segment armed with five spines (Fig. 1A, B, E and F), mesonotum with five pairs of spines on the sides of median longitudinal carina (Fig. 1A and B), anal segment with two strap-shaped bulges on posterolateral margin (Fig. 1E), all femora with distinct pointed serrations (Fig. 1A and B); ♂♂, without spines between the compound eyes, occipital with four spines (Fig. 2C), abdominal tergites II–VIII: each segment armed with three spines (Fig. 2A, B, E and F), all femora with distinct pointed serrations (Fig. 2A and B).

#### Etymology

This specific epithet is derived from Gulinqing Provincial Nature Reserve where it was collected.

## Identification Keys

### Key to females of *Andropromachus* Carl, 1913 worldwide

**Table:** 

1	Abdominal tergites lacking triangular posterolateral tooth	*A.bicolor* (Kirby, 1904) (Brock et al. 2021, Hennemann 2007, Hennemann et al. 2008, Kirby 1904)
–	Abdominal tergites with triangular posterolateral tooth	2
2	All femora unarmed or with indistinct serrations	*A.scutatus* Carl, 1913 (Carl 1913, Hennemann 2007, Hennemann et al. 2008)
–	All femora with distinct serrations	3
3	Between the compound eyes without spine; abdominal tergites II-IV each armed with 4 spines; body length < 40 mm	*A.guangxiense* (Chen & He, 2000) (Chen and He 2000, Chen and He 2008)
–	Between the compound eyes with 2 spines; abdominal tergites II–V each armed with 5 spines; body length > 50 mm	*A.gulinqingensis* sp.n.

### Key to males of *Andropromachus* Carl, 1913 worldwide

**Table:** 

1	Abdominal tergites I–IIX unarmed	*A.bicolor* (Kirby, 1904) (Kirby 1904, Brock et al. 2021, Hennemann 2007, Hennemann et al. 2008)
–	Abdominal tergites I–IIX with a few spines	2
2	Between the compound eyes with 2 spines, occipital with 6 spines	*A.guangxiense* (Chen & He, 2000) (Chen and He 2000, Chen and He 2008)
–	Between the compound eyes without spines; occipital with 4 spines	*A.gulinqingensis* sp. n.

## Supplementary Material

XML Treatment for Andropromachus gulinqingensis

## Figures and Tables

**Table 1.** List of the genus *Andropromachus* of species and distribution.

Species	Female	Male	Distribution	Note
*A.bicolor* (Kirby, 1904)	known	known	N Vietnam	
*A.guangxiense* (Chen & He, 2000)	known	known	SW China: Guangxi Province	
*A.gulinqingensis* **sp.n.**	known	known	SW China: Yunnan Province	
*A.scutatus* Carl, 1913	known	unknown	N Vietnam	Type-species
*A.tonkinensis* (Brunner von Wattenwyl, 1907)	unknown	known	N Vietnam	Loss of type specimen
